# (Poly)phenols and Multiple Sclerosis: Results from an Observational Cross-Sectional Study

**DOI:** 10.3390/antiox14020188

**Published:** 2025-02-06

**Authors:** Monica Guglielmetti, Cinzia Ferraris, Anna Tagliabue, Evelyn Frias-Toral, Eleonora Tavazzi, Alessandro La Malfa, Giacomo Greco, Roberto Bergamaschi, Raynier Zambrano-Villacres, Justyna Godos, Giuseppe Grosso

**Affiliations:** 1 Human Nutrition Center, Department of Public Health, Experimental and Forensics Medicine, University of Pavia, 27100 Pavia, Italy; monica.guglielmetti@unipv.it (M.G.); anna.tagliabue@unipv.it (A.T.); 2 Food Education and Sport Nutrition Laboratory, Department of Public Health, Experimental and Forensics Medicine, University of Pavia, 27100 Pavia, Italy; 3 School of Medicine, Universidad Católica de Santiago de Guayaquil, Av. Pdte. Carlos Julio Arosemena Tola, Guayaquil 090615, Ecuador; 4 Multiple Sclerosis Center, IRCCS Mondino Foundation, 27100 Pavia, Italy; eleonora.tavazzi@mondino.it (E.T.); alessandro.lamalfa02@universitadipavia.it (A.L.M.); giacomo.greco@mondino.it (G.G.); roberto.bergamaschi@mondino.it (R.B.); 5 Escuela de Nutrición y Dietética, Universidad Espíritu Santo, Samborondón 0901952, Ecuador; rzambranovillacres@uees.edu.ec; 6 Department of Biomedical and Biotechnological Sciences, University of Catania, 95123 Catania, Italy; giuseppe.grosso@unict.it; 7 Center for Human Nutrition and Mediterranean Foods (NUTREA), University of Catania, 95123 Catania, Italy

**Keywords:** (poly)phenols, multiple sclerosis, dietary habits

## Abstract

(Poly)phenols are a wide and heterogeneous class of substances with several potential health benefits. Their role in neuroprotection and cognition is still questionable. This study’s scope is to examine the possible association between total and individual (poly)phenol intake, major dietary sources, and the severity of multiple sclerosis (MS) in a cohort of MS patients. Participants’ demographics, physical activity, smoking, and dietary information were collected, alongside clinical parameters including the Expanded Disability Status Score (EDSS), Multiple Sclerosis Severity Score (MSSS), MS phenotype, and current therapy. A validated 110-item food frequency questionnaire (FFQ) was used to assess participants’ habits. The (poly)phenol content of foods was estimated using the Phenol-Explorer database. Data from 106 participants were analyzed. A high intake of vegetables was associated with a 4.6-fold higher probability of mild MS (95% CI: 1.49, 14.28), whereas no association was found for other food and beverage sources. Hydroxycinnamic acids were significantly related to MSSS (OR: 6.55, 95% CI: 2.15, 19.92). Although coffee intake differed significantly between patients with mild and severe MS (90.5 ± 53.9 vs. 59.4 ± 40.8 mL/d, respectively), linear regression analysis did not confirm an association with MSSS. A higher intake of hydroxycinnamic acids and vegetables may impact MS severity. Coffee’s role remains unclear and needs to be further investigated.

## 1. Introduction

(Poly)phenols are a wide and heterogeneous class of natural substances, also ascribed within the phytochemical compounds. They derive from plant secondary metabolism and are characterized by chains of phenolic rings. (Poly)phenols amount to about 8000 different types of molecules [1] with distinct chemical structures, conferring to them distinct pharmacodynamic and pharmacokinetics effects [2,3]. There are several subgroups of (poly)phenols, mainly represented by flavonoids and their main groups (i.e., flavonols, flavan-3-ols, flavanones, anthocyanins, and isoflavones) and non-flavonoid molecules (i.e., phenolic acids, resveratrol, other stilbenes, lignans, tyrosols, and tannins) [4,5]. Being (poly)phenols contained in plant-derived foods and beverages [6], their major dietary sources are fruit and vegetables, coffee, tea, cocoa, and its derivatives (i.e., chocolate), red wine, and curcumin [7,8]. Although much of the evidence regarding the benefits of (poly)phenols comes from in vitro studies and research using pure substances, there is general agreement that these molecules also exert biological activities in humans. In addition to the well-documented antioxidant and anti-inflammatory effects, some recent studies reported the prebiotic action of (poly)phenols on gut microbiota [4,9,10,11]. This made them subjects of several investigations aiming to deepen their potential role in reducing risk factors for chronic non-communicable diseases [4,12,13] and promoting overall health [14]. A recently published study reported beneficial dose-dependent effects of lavender on glucose tolerance, adipose tissue activity, and lipid and lipoprotein metabolism in male C57BL/6 mice [15]. Moreover, some studies have highlighted the beneficial effect of (poly)phenols on depression and Parkinson’s disease risk [16,17]. While the (poly)phenols’ role in cognition and neuroprotection has been evaluated, studies examining their impact on multiple sclerosis (MS) are still scarce.

MS is a pathological condition characterized by neuroinflammation, central nervous system demyelination, and autoimmune activation [18]. Focal inflammation, blood–brain-barrier damage, oxidative stress, and neurodegeneration can determine axonal injury, neuronal loss, and gliosis [19,20,21,22], leading to the formation of characteristic “plaques”. Several motor, cognitive, and sensorial symptoms can follow such basic pathological anatomy alterations, depending on the area affected by demyelination [21,22]. MS derives from the union of genetic, immune, and environmental factors [23,24,25]. Physical activity, smoking habits, and diet have been described as contributing factors to MS development. More recently, the role of the intestinal microbiota has been suggested to potentially play a role in MS pathogenesis [26]. Data from 16S rRNA sequencing revealed that individuals with MS are more likely to have a dysbiotic gut compared to controls [21]. To date, no definitive cure exists for MS. However, disease-modifying therapies (DMTs) can help slow disease progression, reduce the occurrence of new relapses, and prevent the onset of new lesions, making them the standard treatment for MS [27]. Targeting the pathophysiological mechanisms responsible for MS is the main scope of currently available therapies and the goal of ongoing and future research [28]. Several pharmacological and non-pharmacological approaches have been studied, including dietary factors, which may contribute to the amelioration of MS-related outcomes. In fact, the overall diet quality and, more specifically, some of its components can exert both a beneficial or detrimental impact on MS pathogenesis and course [29,30,31,32,33]. The exact mechanisms underlying these effects are still not fully clarified but seem to be related to a combination of indirect pathways, involving inflammation, immune system activation, and some risk of comorbidities (i.e., obesity, lipid profile alterations, and cardiovascular issues) [14,34,35,36]. Concerning (poly)phenols, after undergoing exogenous transformations via the gut microbiota and endogenous transformations in the liver, some of their metabolites have been demonstrated to cross the blood–brain barrier and exert direct anti-inflammatory and antioxidant effects in the central nervous system [37]. Moreover, (poly)phenols have been shown to influence gut microbiota health/eubiosis, hence indirectly regulating the local and systemic immune status [37]. Data retrieved from prospective and longitudinal studies support (poly)phenol’s positive effects on language, memory, and cognition, reporting an inverse association between (poly)phenol consumption and the risk of cognitive impairment [38,39]. The antioxidant and anti-inflammatory effects of the (poly)phenol-rich foods may also be of interest to counteract the pathological processes leading to demyelination and neurodegeneration in MS [8]. Nevertheless, evidence from human studies is scarce. As far as we are aware, to date, the currently available literature data on (poly)phenols and MS mainly derive from studies investigating in depth the mechanistic aspects and pathways [37,40] or specifically focus on single (poly)phenol sources (e.g., coffee, curcumin) [41]. Thus, the present study aimed to investigate the possible association between total (poly)phenol intake, individual (poly)phenol groups, and major dietary sources with MS disease severity in a cohort of people with MS (pwMS) from northern Italy.

## 2. Materials and Methods

This study was designed and carried out by the Human Nutrition and Eating Disorders Research Center from the University of Pavia in collaboration with C. Mondino National Neurological Institute, Pavia, Italy, and the University of Catania. The San Matteo Ethical Committee approved the study procedures (approval number: P-20200064205, date: 5 August 2020), which were conducted according to the Declaration of Helsinki principles. Patients routinely referred to the Multiple Sclerosis Center for follow-up visits were invited to participate in this study. The detailed protocol and procedure explanations are reported in our previous studies [30,42]. MS diagnosis was performed according to McDonald criteria [43]. No restriction was made in the inclusion criteria based on MS phenotype or disability level.

Data retrieved were self-reported by study participants via telephone interviews lasting approximately 30–40 min, conducted due to the COVID-19 pandemic during the initial phases of this study. Information collected included participants’ demographics (age, sex, educational level, marital status) physical activity, smoking habits, and dietary details. Neurological data including Expanded Disability Status Scale (EDSS) scores, Multiple Sclerosis Severity Score (MSSS), MS phenotype, current therapy, and therapy type were gathered by clinical registries and charts.

### 2.1. Demographics, Smoking Habits, and Physical Activity

As far as demographics is concerned, age and sex were collected. Marital status was grouped into the following categories: (i) unmarried and widowed, and (ii) married. Three classes of educational level were created and divided into (i) low (primary/secondary), (ii) medium (high school), and (iii) high (university or above). Smoking habits were arranged as (i) non-smokers, (ii) smokers, and (iii) ex-smokers. The International Physical Activity Questionnaire (IPAQ) [44] was used to assess physical activity habits. It comprises a set of 4 questionnaires (5 activity domains asked independently). Either telephone or self-administered methods are available. For this study, the telephonic interview was chosen. The IPAQ tool evaluates how much time in a week an individual spent being physically active. Particularly, the individual is asked to state how much time (in terms of hours and/or minutes), in the 7 days before completion, they spent sitting (e.g., reading, watching television), and performing moderate physical activity (e.g., gardening, cleaning, bicycling at a regular pace, swimming) or vigorous physical activity (e.g., chopping woods, aerobics, jogging/running, or fast bicycling). The questionnaire also investigates the time spent walking for at least 10 min per day and the job-related physical activity (e.g., transportation, vigorous or moderate activity as part of the work, heavy lifting, digging, heavy construction, or climbing upstairs). Patients were then classified into 3 groups on the basis of their physical activity level (low/moderate/high).

### 2.2. Nutritional Information and (Poly)phenol Content Calculation

During a telephonic interview, participants were asked to indicate their body weight (BW) in kilograms (kg) and height (H) in meters (m). Body Mass Index (BMI) was then derived and classified into underweight (BMI < 18.5 kg/m^2^), normal weight (18.5 ≤ BMI ≤ 24.9 kg/m^2^), overweight (25 ≤ BMI ≤ 29.9 kg/m^2^), and obesity (BMI ≥ 30 kg/m^2^). A validated semi-quantitative food frequency questionnaire (FFQ) [45] investigating the dietary habits of the previous 6 months was administered to each individual by an expert registered dietitian. Nine frequency options were available for each of the 110 items (“never”, “once a month”, “twice a month”, “once a week”, “2–3 times/week”, “4–5 times/week”, “once a day”, “2–3 times/day”, “4–5 times/day”). A reference portion size was given for some foods and, if the portion commonly consumed by the participant did not correspond to the one indicated in the FFQ, the frequency of consumption was modified accordingly. The standard portion size was then multiplied by the reported frequency of consumption and then converted into daily intake (in grams or milliliters). When possible, the dietitian asked each participant whether the specific food was consumed raw, cooked, or preserved (i.e., ready to eat). The daily intake of each food item was multiplied for its (poly)phenol content and retrieved using the Phenol-Explorer database. This procedure was repeated for (poly)phenol subclasses and for specific food and beverage sources. Study participants were categorized into low and high (poly)phenol consumers according to the median intake of (poly)phenols, registered for the whole sample. Based on the distribution of (poly)phenol consumption in the study sample, participants were divided into “low” and “high” (poly)phenol intake according to the group-specific median cut-off values.

### 2.3. Expanded Disability Status Score and Multiple Sclerosis Severity Score

The neurological disability of MS patients was quantified by the Expanded Disability Status Scale (EDSS), which rates seven neurological domains [Visual, Brainstem, Pyramidal (motor), Cerebellar (coordination), Sensory, Cerebral, and Bowel/bladder] in the context of a standard neurological examination. Ambulation scoring concludes the evaluation. The final EDSS was assigned according to the scores attributed to the single neurological systems [46]. The clinical impact of MS was calculated by applying the Multiple Sclerosis Severity Score (MSSS) [47]. The MSSS represents the severity of MS at a given time; it is calculated using an algorithm that adjusts the EDSS according to the corresponding disease duration. Herbert’s severity grading [48], based on the different values of the MSSS, was applied to categorize the participants according to their level of disability. Herbert divided MS patients into six approximately equipopulated groups of disability. Patients with an MSSS < 1.7 would be classified as exhibiting mild MS; patients with MSSS values ranging between 1.7 and 3.4 would be defined as exhibiting moderate MS; patients with an MSSS from 3.4 to 5.0 would be defined as being affected by intermediate MS; an MSSS ranging between 5.0 and 6.7 corresponds to accelerated MS; MSSS scores of 6.7 to <8.3 indicate an advanced MS; and values of MSSS above 8.3 stand for an aggressive MS. Based on the distribution of disease severity in the study sample, participants were categorized as “mild” (mean MSSS values < 1.7) and “moderate to high” MS (mean MSSS values > 1.7).

### 2.4. Statistical Analysis

Mean and standard deviations (SDs) were adopted to describe continuous variables, with Student’s *t*-test used to explore differences between groups. Frequencies of occurrence and percentages were used to describe categorical variables, with the Chi-squared test used to determine the differences between groups. The association between (poly)phenol intake and MS severity was assessed through logistic regression analysis, providing odds ratios (ORs) and the respective 95% confidence intervals (CIs). Unadjusted and multivariate models adjusted for possible confounding factors (energy intake, age, sex, BMI, educational status, smoking status, and physical activity level) were performed. *p*-values were based on two-sided tests. The significance level was set at 5%. SPSS 27 (SPSS Inc., Chicago, IL, USA) software version 29 was used for the statistical analysis.

## 3. Results

Enrollment included 130 pwMS, but a final sample of 106 patients was analyzed after the exclusion of some participants due to insufficient data (n = 19) and refusal to participate in the telephone interview (n = 5).

The background characteristics of the participants are summarized in Table 1. Both in the low vs. high (poly)phenol consumption groups, most of the participants were females (60.5% and 77.8%, respectively), had a medium educational level (52.8% and 46.3%, respectively), and were in the normal-weight class of BMI (67.9% and 64.8%, respectively). Low–medium physical activity was the most frequent among the two groups, representing 75.5% and 70.3% of the sample. Never smokers, as well, comprised 67.9% and 55.6% of the participants. As far as neurological information is concerned, the mean MSSS was 2.9 ± 2.5 in the low-consumption group and 3.4 ± 2.4 in the high-consumption one. Secondary progressive MS was the most common type of MS, with 75.5% and 68.5% of the participants within this category. Proportions of 66.0% and 66.7% of the participants of the two groups were undergoing MS medications, with the moderate-efficacy ones being the most used (68.6% and 80%, respectively). No significant differences were found in demographic, lifestyle, or clinical variables between the low- and high-(poly)phenol-consumption groups.

**Figure 1:**
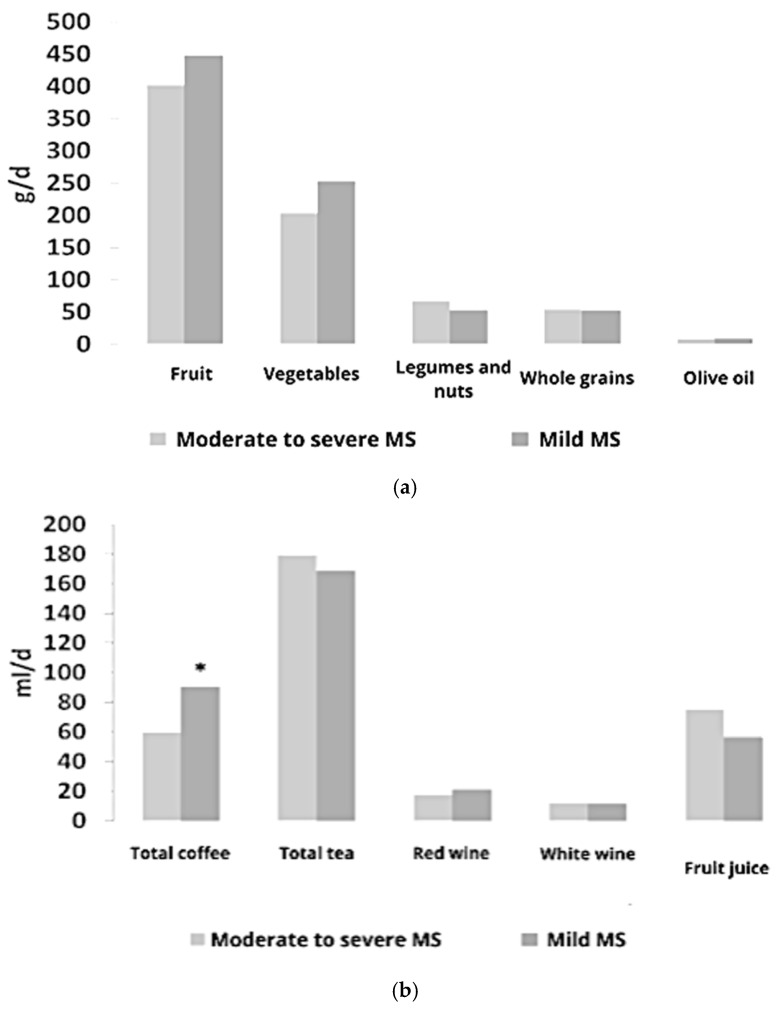
Mean intake of dietary sources of (poly)phenols (foods and beverages) in the two study groups. (**a**) represents the daily intakes (g/d) of (poly)phenols food sources, while (**b**) describes their beverage sources. Significant different intakes were reported using an asterix *.

The mean consumption of (poly)phenol-rich foods and beverages according to MS severity is shown in Figure 1a and Figure 1b, respectively. No significant differences between groups were detected with the exception for (espresso) coffee consumption, which was higher in patients with mild compared to moderate-to-severe MS symptoms (90.5 ± 53.9 and 59.4 ± 40.8 mL/d, respectively).

However, when testing the association between major food and beverage sources of (poly)phenols and the likelihood of exhibiting mild MS (compared to moderate-to-severe forms), only high vegetable intake was associated with a 4.6-fold higher probability of mild MS (95% CI: 1.49, 14.28), whereas no significant association was found among the other food and beverage groups (Table 2).

The associations between total and major (poly)phenol subclasses and mild MS are presented in Table 3. The regression model revealed no significant findings, except for hydroxycinnamic acids (HCAs), which demonstrated a strong association with mild MS (OR 6.55, 95% CI: 2.15, 19.92).

## 4. Discussion

This cross-sectional study aimed to evaluate the possible relationship between (poly)phenol intake and the severity of MS. Our findings revealed a significant difference in coffee intake between patients with mild and more severe MS symptoms. However, only a higher intake of specifically HCA (among (poly)phenols) and vegetables (among food groups) was independently related to MS severity.

Previous studies have investigated the potential role of vegetable consumption in MS, describing it as having beneficial effects on this condition [49,50]. A study evaluating diet quality in a sample of 2047 pwMS reported that greater vegetable and fruit consumption was associated with lower disability levels and a better quality of life [51]. Another multicenter study involving 219 pediatric pwMS or Clinically Isolated Syndrome (CIS) found that each additional portion of vegetable consumption was linked to a 50% reduction in the risk of MS [52]. Moreover, a pilot study on 20 Italian individuals with MS explored the effect of a 1-year intervention with a high-vegetable/low-protein diet compared to a Western diet. This intervention positively influenced several MS-related aspects, including gut microbiota, relapse rate, and inflammatory markers, reporting a decrease in IL-17-producing CD4+ T lymphocytes, increased abundance of the *Lachnospiraceae* family, and reduced relapse rate in the high-vegetable/low-protein group [53]. These effects might be potentially attributed to several factors. First, vegetables are rich in various beneficial compounds such as fiber, minerals, vitamins, and (poly)phenols [54]. Second, intestinal microbiota metabolism and fermentation may play a relevant role. Short-chain fatty acids (SCFAs), produced during intestinal fermentation, can reinforce the intestinal barrier, lessen the production of pro-inflammatory molecules, and, in general, have immunomodulatory properties [55]. Another possible contribution might derive from specific compounds contained in vegetables, such as HCA content. In the present study, HCA intake was found to be associated with a 6.5-fold increase in the probability of exhibiting mild MS. HCAs are one of the most widely diffused (poly)phenols in nature and are present in great amounts in vegetables, fruits, cocoa, wine, tea, and coffee [56]. Their intake is extremely variable within individuals, depending on their age, the food sources, and the specific HCA subgroup considered [57,58]. HCAs, as (poly)phenols in general, present numerous beneficial effects on human health [56,59]. These compounds have demonstrated several potential biological activities on humans, such as antitumoral [60], antihypertensive [61], neuroprotective [62], antioxidant [63,64], antimicrobial [65], and anti-inflammatory properties [66,67]. Among these, the antioxidant and anti-inflammatory ones deserve special attention concerning MS. For example, the phenolic hydroxyl group in HCAs is fundamental for their antioxidant effects as it helps in stabilizing radical oxygen and nitrogen species by reacting with them [68]. Another HCA compound, caffeic acid, may act as a scavenger of nitric oxide and has iron-reducing effects [63,64,69]. The anti-inflammatory activities are linked to different mechanisms, including the capacity of chlorogenic, caffeic, and ferulic acids to down-regulate the LPS-induced expression of nitric oxide synthase [70]. Other possible mechanisms of action involve the aforementioned antimicrobial and prebiotic effects, which support the concept of “duplibiotics”, a bidirectional relationship between (poly)phenols and the intestinal microbiota [71]. Except for small amounts of flavonoids, which are absorbed in the small intestine, the majority of dietary (poly)phenols reach the large intestine, where they are metabolized [72,73,74]. The persistence of (poly)phenols (especially the non-extractable ones) and their metabolites in the bowel has a positive impact on the colon health and its microbiota. The intestinal bacteria, in a dysbiotic condition, can alter metabolites and gut hormone production and release [75], directly interact with specific brain cells, and modify neural and immune signals [76,77]. All these alterations can have systemic effects, including increased blood–brain barrier permeability, immune system dysregulation, and autoimmune demyelination [26]. Dysbiosis can also be associated with inflammation and the subsequent increase in Th17 cells and pro-inflammatory cytokine expression [78]. (Poly)phenols can counteract this situation, contributing to strengthening the intestinal barrier by favoring the expression of tight junction proteins, and mitigating inflammation [79]. 

In our study, coffee consumption was statistically different between those with high and low (poly)phenol consumption. Similar findings have been reported in a case–control study that associated black tea (OR = 0.20), green tea (OR = 0.29), and coffee (OR = 0.07) with a lower risk of MS [80]. An inverse relationship between coffee, fish, and alcohol consumption and MS probability was also described in another study [81]. Hedström et al. [82,83] also conveyed that higher coffee consumption was associated with lower MS probability. According to a meta-analysis, this association can also be extended to neurological diseases in general, as high caffeine doses were found to be preventive of these conditions [84]. In contrast, other studies reported no effect of caffeine (or coffee) on MS [82,83]. Coffee’s impact can be mediated by its high (poly)phenol content [80], caffeine’s stimulatory effect on the central nervous system, or other unexplored mechanisms.

This study has some limitations that need to be addressed when considering the reported findings. First, the observational design only allows putative conclusions about the causality between (poly)phenol intake and MS severity. Moreover, a broader population, possibly from different regions, would have provided a more representative sample. The use of an FFQ introduces the possibility of over- or under-reporting due to recall and social desirability biases and does not fully distinguish between fresh or preserved foods, possibly impacting on (poly)phenol esteem. Differences in the estimation of polyphenol content can also result from the species of plants used, growth conditions, etc., which were not evaluated in this paper. In addition, it is worth emphasizing that to achieve more tangible results, further work is necessary, taking into account personal contact with the patient and, more precisely, assessing the content of polyphenols in consumed food. Finally, although many variables were accounted for when considering the possible confounding factors, we cannot exclude the existence of unmeasured confounders (e.g., inflammation parameters, total polyphenols, antioxidants, or oxidative stress biomarkers).

## 5. Conclusions

In conclusion, a higher intake of HCA and vegetables may play a role in MS gravity. However, the role of coffee, a major source of HCA, remains unclear. These findings and their underlying mechanisms need to be further explored by case–control or intervention studies.

## Figures and Tables

**Table 1:** Baseline characteristics of the study sample according to total (poly)phenol intake (*n* = 106).

Total (Poly)phenol Intake			
	Low	High	*p*-Value
Age, mean (SD)	49.6 (12.2)	51 (11.1)	0.538
Sex, *n* (%)			0.051
Men	21 (39.6)	12 (22.2)	
Women	32 (60.4)	42 (77.8)	
Educational level, n (%)			0.792
Low	10 (18.9)	12 (22.2)	
Medium	28 (52.8)	25 (46.3)	
High	15 (28.3)	17 (31.5)	
BMI, n (%)			0.516
Normal-weight	36 (67.9)	35 (64.8)	
Overweight	15 (28.3)	14 (25.9)	
Obese	2 (3.8)	5 (9.3)	
Physical activity level, n (%)			0.764
Low	23 (43.4)	20 (37.0)	
Medium	17 (32.1)	18 (33.3)	
High	13 (24.5)	16 (29.6)	
Smoking status, n (%)			0.304
Never	36 (67.9)	30 (55.6)	
Current	7 (13.2)	13 (24.1)	
Former	10 (18.9)	11 (20.4)	
MSSS, mean (SD)	2.9 (2.5)	3.4 (2.4)	0.411
Disease course, n (%)			0.697
Secondary progressive	11 (20.8)	15 (27.8)	
Relapsing-remitting	40 (75.5)	37 (68.5)	
Primary progressive	2 (3.8)	2 (3.7)	
Current therapy, n (%)			0.945
No	18 (34.0)	18 (33.3)	
Yes	35 (66.0)	36 (66.7)	
Therapy type, n (%)			0.274
Moderate efficacy	24 (68.6)	28 (80.0)	
High efficacy	11 (31.4)	7 (20.0)	

BMI = Body Mass Index; MSSS = Multiple Sclerosis Severity Score.

**Table 2:** Association between major food and beverage sources of (poly)phenol intake and mild multiple sclerosis (as compared to moderate-to-high).

Mild Multiple Sclerosis, OR (95% CI) *		
	Low Consumption	High Consumption
(Poly)phenol food sources		
Fruit	1	1.31 (0.46, 3.77)
Vegetables	1	4.61 (1.49, 14.28) ^a^
Legumes and nuts	1	1.10 (0.43, 2.82)
Whole grains	1	1.22 (0.47, 3.14)
Olive oil	1	1.58 (0.57, 4.41)
(Poly)phenol beverage sources		
Tea	1	0.61 (0.23, 1.64)
Coffee (espresso/stovetop)	1	1.85 (0.29, 11.99)
White wine	1	2.20 (0.82, 5.90)
Red wine	1	1.40 (0.55, 3.58)
Juices	1	0.60 (0.23, 1.57)

* Adjusted for energy intake (continuous, kcal/d), age (continuous, years), sex (male, female), BMI (normal, overweight, obese), educational status (low, medium, high), smoking status (never, current, former), and physical activity level (low, medium, high). OR = odds ratio; CI = confidence interval. ^a^ *p*-value < 0.001.

**Table 3:** Association between total (poly)phenol and major subclass intake and mild multiple sclerosis (as compared to moderate-to-high).

Mild Multiple Sclerosis, OR (95% CI) *		
	Low Consumption	High Consumption
Total (poly)phenols	1	1.48 (0.53, 4.16)
Flavonoids	1	1.26 (0.46, 3.44)
Phenolic acids	1	1.49 (0.52, 4.26)
Stilbenes	1	1.93 (0.73, 5.11)
Lignans	1	0.57 (0.22, 1.49)
Anthocyanins	1	1.54 (0.54, 4.36)
Flavonols	1	1.10 (0.39, 3.08)
Flavanols	1	0.77 (0.29, 2.04)
Flavanones	1	0.57 (0.22, 1.48)
Flavones	1	1.17 (0.42, 3.23)
Hydroxycinnamic acids	1	6.55 (2.15, 19.92) ^a^
Hydroxyphenylacetic acid	1	0.97 (0.36, 2.59)

* Adjusted for energy intake (continuous, kcal/d), age (continuous, years), sex (male, female), BMI (normal, overweight, obese), educational status (low, medium, high), smoking status (never, current, former), and physical activity level (low, medium, high). OR = odds ratio; CI = confidence interval. ^a^ *p*-value < 0.001.

## Data Availability

The data presented in this study are available on request from the corresponding author due to privacy reasons.
